# Immune-Stimulatory Effects of Curcumin on the Tumor Microenvironment in Head and Neck Squamous Cell Carcinoma

**DOI:** 10.3390/cancers13061335

**Published:** 2021-03-16

**Authors:** Charlotte Kötting, Linda Hofmann, Ramin Lotfi, Daphne Engelhardt, Simon Laban, Patrick J. Schuler, Thomas K. Hoffmann, Cornelia Brunner, Marie-Nicole Theodoraki

**Affiliations:** 1 Department of Otorhinolaryngology, Head and Neck Surgery, University of Ulm, 89070 Ulm, Germany; charlotte.koetting@uni-ulm.de (C.K.); linda.hofmann@uni-ulm.de (L.H.); daphne.engelhardt@uniklinik-ulm.de (D.E.); simon.laban@uniklinik-ulm.de (S.L.); patrick.schuler@uniklinik-ulm.de (P.J.S.); t.hoffmann@uniklinik-ulm.de (T.K.H.); cornelia.brunner@uniklinik-ulm.de (C.B.); 2 Institute for Clinical Transfusion Medicine and Immunogenetics Ulm, German Red Cross Blood Services Baden-Württemberg-Hessen, 89081 Ulm, Germany; r.lotfi@blutspende.de; 3 Institute for Transfusion Medicine, University Hospital Ulm, 89081 Ulm, Germany

**Keywords:** head and neck squamous cell carcinoma, NF-κB, curcumin, Poly I:C, NF-κB inhibitors, epithelial to mesenchymal transition, modulation of tumor microenvironment

## Abstract

**Simple Summary:**

Head and neck squamous cell carcinoma has been shown to downregulate the host’s antitumor immune response as well as inherent anticancer immunity, inter alia, via increased activation of nuclear factor kappa of activated B-cells (NF-κB). The aim of this study is to examine curcumin’s effects on certain pro- and antitumoral chemokines via NF-κB, as well as the combined effects of curcumin and toll-like receptor 3 agonist Poly I:C on NF-κB and regulatory T-cell attraction. Furthermore, we compare curcumin with established NF-κB inhibitors caffeic acid phenethyl ester and BAY 11-7082. We demonstrate that curcumin has immune-modulating effects, with potent inhibition of the regulatory T-cell-attracting effects of Poly I:C. Therefore, curcumin presents an adjuvant that not only improves the effects of established therapies but also holds the potential to reduce negative side effects in tumor entities with increased NF-κB activation.

**Abstract:**

Curcumin is known to have immune-modulatory and antitumor effects by interacting with more than 30 different proteins. An important feature of curcumin is the inhibition of nuclear factor kappa of activated B-cells (NF-κB). Here, we evaluate the potential of curcumin to reverse the epithelial to mesenchymal transition (EMT) of head and neck squamous cell carcinoma (HNSCC) cells as a part of tumor escape mechanisms. We examined the impact of curcumin on the expression of different pro- and antitumoral chemokines in ex vivo HNSCC tumor tissue and primary macrophage cultures. Further, we evaluated the combinatorial effect of curcumin and toll-like receptor 3 (TLR3) agonist Poly I:C (PIC) on NF-κB inhibition and regulatory T-cell (Treg) attraction. Mesenchymal markers were significantly reduced in cancer specimens after incubation with curcumin, with simultaneous reduction of key transcription factors of EMT, Snail, and Twist. Furthermore, a decrease of the Treg-attracting chemokine CCL22 was observed. Additionally, curcumin-related inhibition of NF-κB nuclear translocation was evident. The combination of PIC with curcumin resulted in further NF-κB inhibition, whereas PIC alone contrarily resulted in NF-κB activation. Furthermore, curcumin was more effective in inhibiting PIC-dependent NF-κB activation and Treg attraction compared to known NF-κB inhibitors BAY 11-7082 or caffeic acid phenethyl ester (CAPE). The presented results show, for the first time, the immune-modulating effects of curcumin in HNSCC, with potent inhibition of the Treg-attracting effects of PIC. Hence, curcumin presents a promising drug in cancer therapy as a supplement to already established treatments.

## 1. Introduction

Head and neck squamous cell carcinomas (HNSCCs) belong to the seven most frequent cancers worldwide [1,2]. Despite progress in terms of chemo-, radio- and targeted therapies, recurrences and distant metastases occur in up to 25–30% of patients [3,4], with a poor five-year survival rate [5]. HNSCCs have been demonstrated to suppress inherent anticancer immunity and to downregulate signals of the hosts’ antitumor immune response, which conversely results in tumor growth and progression [6,7]. The tumor microenvironment (TME), which describes the immediate cancer surrounding, is known to play an important role in terms of tumor progression [8]. Containing immune cells such as T-cells, macrophages, dendritic cells (DCs), myeloid-derived suppressor cells (MDSCs) as well as various chemokines, [6,8,9], the TME as a dynamic construct, allowing a quick response to immune reaction or treatment, inter alia, and resulting in immune suppression and angiogenesis [8]. Regulatory T-cells (Tregs) and MDSCs play a decisive role in the TME by suppressing the antitumor immune response and favoring tumor progression and metastasis. Reports show a correlation between high Treg levels and poor overall survival rates [10,11]. Cytotoxic T-cells (CTLs), on the other hand, have the opposite effect by eradicating tumor cells with perforin and FAS-mediated mechanisms, resulting in an antitumor immune response [11]. Whether Tregs or CTLs predominate in the TME depends on different tumor-cell- and macrophage-released chemokines [10]. While CXCL12 and CCL22 lead to Treg and MDSC attraction, resulting in a downregulation of the antitumor immune response [12,13], CCL5 and CXCL10 lead to CTL attraction and immune response [14]. Moreover, macrophages not only release TME-affecting chemokines but also create an inflammatory environment and lead to apoptosis, especially in the early stages of tumor degeneration [15]. Tumor-associated macrophages in HNSCC patients are associated with poor prognosis [16,17,18] and are known to overexpress toll-like receptor 3 (TLR3) [19].

Referring to metastasis, another influencing factor is epithelial to mesenchymal transition (EMT). EMT plays a physiological role in tissue regeneration and wound healing, whereas, under nonphysiological conditions, it leads to increased mobility, favoring metastasis and loss of organ function [20,21]. EMT can be induced through master regulators such as Twist and Snail, resulting in decreased epithelial markers such as E-cadherin and upregulated mesenchymal markers like Vimentin or N-cadherin [22,23]. Snail expression is especially augmented by activation of the nuclear factor kappa of activated B-cells (NF-κB) signaling pathway. NF-κB, on the other hand, is often overexpressed in many tumor entities and is moreover associated with a malignant phenotype in HNSCCs [24]. By affecting cell survival, tumorigenesis, proliferation, migration, and metastasis, NF-κB constitutes a key factor in tumor progression, surveillance, and regression [24,25]. This is one reason why curcumin, a component of “Curcuma longa”, commonly known as turmeric [26,27,28], has gained increasing interest in recent years. Previous research has verified its antioxidant, antimicrobial, anti-inflammatory, antiangiogenic, antimutagenic, protective, and preventive effects regarding various diseases and confirmed its chemosensitizing and chemopreventive effects [26,27,28]. One important effect of curcumin is the inhibition of NF-κB [21,29] by preventing the retention of p65 [30] and by inhibition of the NF-κB/Snail signaling cascade [31], resulting in reduced tumor progression. In contrast, Poly I:C (PIC), a synthetically produced double-strand RNA, is a potent TLR3 agonist and cytoplasmic helicase activator [32]. Ligands for TLR3 have been evaluated extensively in clinical trials [33,34]. However, outcomes remain comparatively poor, and current studies are mainly focused on glioblastoma [35]. In previous studies, we analyzed upstream signaling pathways and showed that PIC mainly activates NF-κB via TLR3 and cytoplasmic helicases, while induction of the type I interferon pathway was induced by TLR3 signaling only. In contrast, when the selective TLR3 agonist Rintatolimod was used, only targeted activation of the TLR3-dependent pathway was visible, with similar induction of type I interferons but less NF-κB activation [32]. Since NF-κB activation is accompanied by tumor-favoring effects [25] and curcumin presents as a selective NF-κB inhibitor [29], we expected that curcumin might be able to minimize the negative effects induced by PIC, thereby resulting in an exclusive activation of the desired type I interferon pathway. Here, we investigate the potential of curcumin to reduce the undesired NF-kB activation by PIC and compare the downstream effects with commonly known NF-κB inhibitors such as caffeic acid phenethyl ester (CAPE) or BAY 11-7082 [36,37].

## 2. Results

### 2.1. Confirmation of Epithelial to Mesenchymal Transition In Vitro

**Figure 1:**
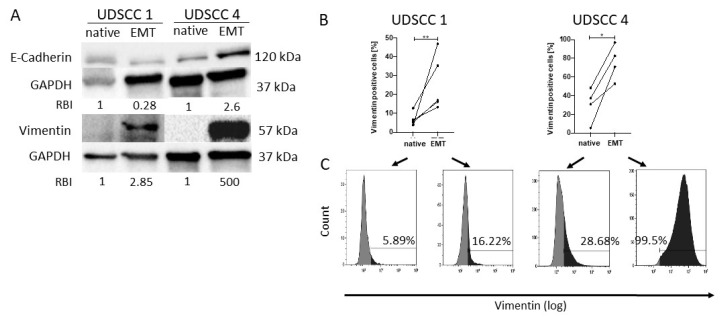
Confirmation of epithelial to mesenchymal transition (EMT) induction. UDSCC1 and UDSCC4 cells were treated with StemXVivo EMT-inducing media supplement for 5 days to induce EMT. (**A**) Western blots were performed for detection of E-cadherin and vimentin expression of native and EMT-induced UDSCC1 and UDSCC4 cell lines. Note the increased expression of vimentin in both cell lines after EMT induction, while E-cadherin expression was increased in UDSCC4 cells but decreased in UDSCC1 cells. Relative band intensities (RBIs) were calculated between E-cadherin and GAPDH or vimentin and GAPDH. The blot is representative for *n* = 3. Original blots can be found in Figure S1. (**B**) Flow cytometry of native and EMT-induced cells revealed significantly higher vimentin levels in EMT cells. *P*-values were determined with the Mann–Whitney test, with * *p* < 0.05, ** *p* < 0.01. (**C**) Representative flow cytometry histograms depicting vimentin expression of native and EMT-induced UDSCC1 and UDSCC4 cell lines. Note that Vimentin expression was increased after treatment with StemXVivo EMT-inducing media supplement, especially for UDSCC4.

University of Düsseldorf squamous cell carcinoma 1 (UDSCC1) and UDSCC4 cell lines were treated with StemXVivo EMT-inducing media supplement for 5 days. Confirmation of EMT induction was performed by Western blot analysis and intracellular flow cytometry for vimentin and E-cadherin. In contrast to E-cadherin, which showed a decrease in UDSCC1 but an increase in UDSCC4 after treatment, uniform results were noticed for vimentin. An increased expression of the mesenchymal marker vimentin was visible in both cell lines after treatment with the EMT cocktail in Western blots (Figure 1A) and flow cytometry (Figure 1B,C), with an especially strong increase in the UDSCC4 cell line.

### 2.2. Curcumin-Dependent Reversion of EMT

**Figure 2:**
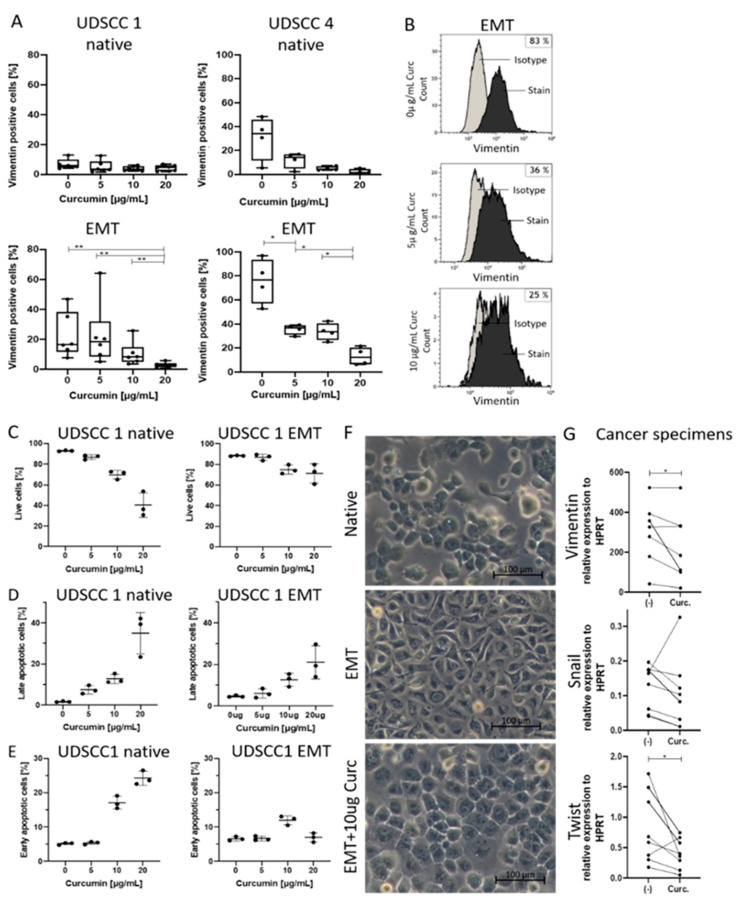
(**A**–**E**) Native and EMT-induced UDSCC1 or UDSCC4 cells were treated with indicated concentrations of curcumin (0, 5, 10, or 20 µg/mL) for 48 h. Then, vimentin expression was analyzed by flow cytometry (**A**,**B**), and apoptosis was assessed by annexin/propidium iodide (PI) assay (**C**–**E**). (**A**) EMT-induced cell lines showed significantly decreased vimentin expression with increased concentrations of curcumin; *n* = 4 (UDSCC4 EMT), *n* = 6 (UDSCC1 EMT and UDSCC4 native), *n* = 7 (UDSCC1). Bars represent minimum to maximum, with a line at the mean. (**B**) Representative flow cytometry histograms depicting vimentin expression of EMT-induced UDSCC4 cells after incubation with curcumin. (**C**) The amount of live UDSCC1 cells was especially reduced for native cells with increased concentrations of curcumin. (**D**) Late UDSCC1 apoptotic cells were detected for native cells with 20 µg/mL curcumin; effects on EMT-induced cells were less prominent. (**E**) The number of early apoptotic cells was higher with increased curcumin concentrations, especially with 20 µg/mL. Effects were more pronounced for native cells. *n* = 3. Bars represent minimum to maximum with a line at the median. (**F**) Native UDSCC cells showed typical epithelial characteristics such as rounded cell bodies (top), while EMT-induced cells showed mesenchymal remodeling with its signifying protrusions (middle). We observed that EMT-induced cells incubated with 10 µg/mL curcumin (Curc.) were able to convert mesenchymal characteristics into epithelial ones (bottom). (**G**) Evaluation of mesenchymal markers (Vim/Snail/Twist) in cancer specimens by qRT-PCR after incubation with the negative control (-) or 10 µg/mL curcumin (Curc.) for 24 h. mRNA levels are shown as relative expression to the normalization control HPRT. *n* = 9, each dot represents an individual tumor sample. *P*-values in (**A**) and (**G**) were determined with the Mann–Whitney test, with * *p* < 0.05, ** *p* < 0.01.

Curcumin is known for its effects on converting EMT back into mesenchymal epithelial transition (MET) [21]. To determine the optimal concentration of curcumin that induces EMT to MET transition in our assay system, native and EMT-induced UDSCC1 and UDSCC4 cells were incubated with different concentrations of curcumin, and their vimentin expression was analyzed by flow cytometry (Figure 2A,B). The greatest impact on the reversion of vimentin expression, especially in EMT-induced cells, was visible with 10 and 20 µg/mL curcumin (Figure 2A,B). However, annexin/ propidium iodide (PI) apoptosis assay showed a decreased number of live cells in correlation with increased concentrations of curcumin, especially with 20 µg/mL (Figure 2C). These effects were greater for native than for EMT-induced cells. In line with these findings, the number of late apoptotic cells was highest for 20 µg/mL, especially in native but also in EMT-induced cells (Figure 2D). Similar effects on early apoptotic cells were only detected for native cells, while EMT-induced cells showed no notable differences between the different concentrations (Figure 2E). Importantly, the apoptotic rate was not influenced by the DMSO concentration as this was kept constant for all treatment conditions and included in the negative controls. Based on these results, 10 µg/mL curcumin was chosen as the optimal concentration for further experiments.

EMT reversion was not only detected as changes of structural proteins but also as changes in cell morphology. After treatment with 10 µg/mL curcumin, the spindle-shaped EMT-induced cells, with typical mesenchymal protrusions, converted back into cuboid-shaped cells, a distinctive epithelial feature with a similar appearance to native cancer cells (Figure 2F).

To further verify EMT reversion in an ex vivo system, cancer specimens were treated with 10 µg/mL curcumin, and the mRNA levels of mesenchymal markers were analyzed by qRT-PCR. Incubations with curcumin resulted in a significant decrease in vimentin and Twist gene expression, with similar results visible for Snail expression, although not significant (Figure 2G).

### 2.3. The Effect of Curcumin on Chemokine Expression in Ex Vivo Tumor Tissues

To analyze the impact of curcumin on the TME, ex vivo tumor tissues and macrophage cultures were incubated with curcumin, TLR3 ligand PIC, or the combination of both, and supernatants were examined by ELISA with regard to the release of CTL-attracting chemokines CXCL10 and CCL5 and Treg-attracting chemokine CCL22. Due to the ability of curcumin to inhibit NF-κB [29] and the capability of PIC to activate NF-κB as well as the desired type I interferon pathway [32], we implemented incubations with the combination of curcumin and PIC to see if an increased activation of the desired signaling pathway via type I interferon can be noticed.

**Figure 3:**
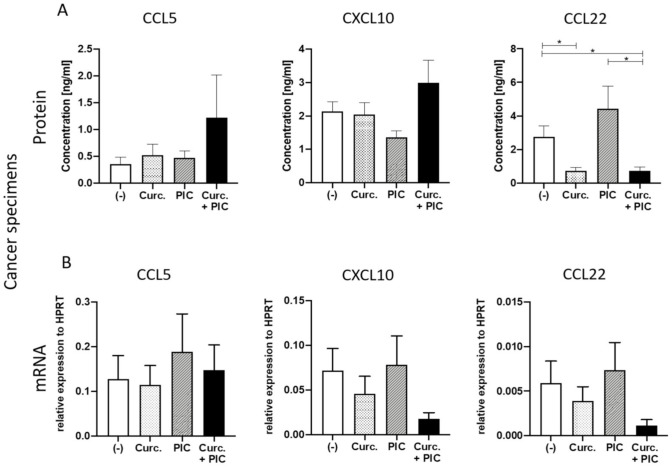
Chemokine expression in ex vivo tumor tissues. (**A**) Detection of chemokines CCL5, CXCL10, and CCL22 in supernatants of cancer specimens after incubation with the negative control (-), 10 µg/mL curcumin (Curc.) and/or PIC for 24 h. Results were determined via ELISA. *n* = 5. (**B**) Evaluation of chemokine expression (CCL5/CXCL10/CCL22 in cancer specimens by qRT-PCR after incubation with the negative control (-), curcumin (Curc.) and/or PIC for 24 h. mRNA levels are shown as relative expression to the normalization control HPRT. *n* = 9. Bars represent mean with standard error of mean (SEM). *P-*values were determined with the Mann–Whitney test, with * *p* < 0.05.

As shown in Figure 3A, chemokine levels of CCL5 and CXCL10 were remarkably increased in supernatants of tumor tissues after combined incubation with curcumin and PIC. Curcumin significantly decreased CCL22 levels in supernatants of tumor samples compared to the untreated controls. Importantly, the high levels of CCL22 in supernatants of PIC-treated tumor samples were significantly decreased upon combination with curcumin. Figure 3B shows gene expression levels of CCL5, CXCL10, and CCL22, with a similar decrease in CCL22 after the combination of PIC with curcumin, compared to PIC alone. Results for CCL5 and CXCL10 were not as clear as in protein levels (Figure 3B). A nonsignificant decrease in CXCL10 mRNA levels is visible from increased protein concentrations. This may be due to the already-visible degradation of mRNA by lasting protein expression.

### 2.4. Effects of Curcumin on Chemokine Expression in Macrophage Cultures

**Figure 4:**
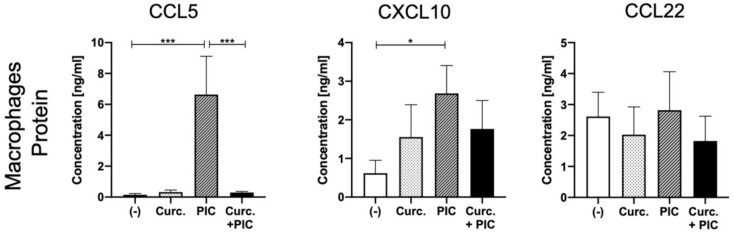
Chemokine expression in macrophage cultures. Detection of chemokines CCL5, CXCL10, and CCL22 from supernatants of macrophage cultures after incubation with the negative control (-), 10 µg/mL curcumin (Curc.) and/or PIC for 24 h. Results were determined via ELISA. *n* = 7. Bars represent mean with SEM. *P-*values were determined with the Mann–Whitney test, with * *p* < 0.05, *** *p* < 0.001.

Similar to ex vivo tumor tissues, macrophage cultures were treated with curcumin, PIC, or the combination of both, and supernatants were analyzed by ELISA. CCL5 and CXCL10 levels were significantly elevated after incubation with PIC, whereas no differences were visible in CCL22 expression. While the combination of curcumin with PIC led to a significantly lower expression of CCL5 compared to PIC alone, no significant differences were visible with CXCL10 and CCL22 (Figure 4).

### 2.5. Curcumin Inhibits the Migratory Potential of Treg

**Figure 5:**
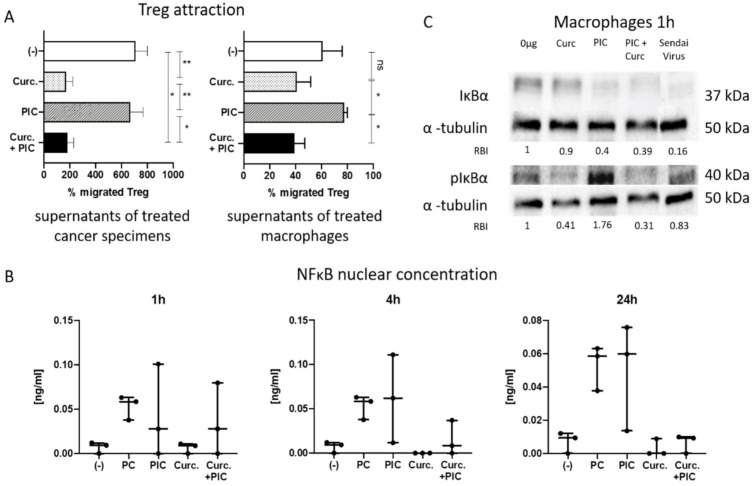
(**A**) Regulatory T-cell (Treg) attraction. Cancer specimens and macrophages were incubated with adjuvants for 24 h. After incubation, supernatants were harvested, and a migration assay was performed for 1.5 h. Flow cytometry was used to count the migrated cells. A decreased amount of migrated Treg can be seen after 10 µg/mL curcumin (Curc.) or 10 µg/mL curcumin + PIC incubation, especially for supernatants of the cancer specimens. (-) indicates the negative control. *n* = 5. Bars represent mean with SEM. *P*-values were determined with the Mann–Whitney test, with * *p* < 0.05, ** *p* < 0.01; ns = not significant. (**B**) Nuclear concentration of NFκB in macrophages after incubation with adjuvants for 1, 4, and 24 h. Note the increased NFκB nuclear concentration in samples treated with the positive control (PC) or PIC in contrast to almost no activation with curcumin (Curc., 10 µg/mL). Treatment with curcumin and PIC shows a clear decrease of NFκB nuclear concentration after 4 and 24 h of incubation. (-) indicates the negative control. Results were determined via ELISA. *n* = 3. Bars represent minimum to maximum with a line at the mean. (**C**) Western blot for IκBα, pIκBα and α–tubulin after 1 h of macrophage treatment as indirect evidence for nuclear translocation of NFκB, showing a decreased expression of IκBα after incubation with PIC and an increased amount of pIκBα as a marker of NFκB nuclear translocation. Although treatment with PIC and curcumin shows only a slight increase in IκBα compared to PIC alone, a decreased expression of activated IκBα (pIκBα) is clearly visible. Relative band intensities (RBIs) were calculated between IĸBα and α-tubulin or pIĸBα and α-tubulin. The blot is representative for *n* = 3. Original blots can be found in Figure S1. The α-tubulin loading controls for IĸBα and pIĸBα shown in (**C**) are identical to the α-tubulin loading controls shown in Figure 6B since they are part of the same original blots; the figures are organized as shown to better align with the data persented in the results section.

Migration assays with CD39+CD4+ Treg towards supernatants of treated macrophages or cancer specimens were performed to evaluate curcumin’s potential to inhibit Treg migration. Curcumin treatment of cancer specimens or macrophages significantly reduced Treg attraction towards the respective supernatants. Moreover, PIC-induced Treg attraction was significantly reversed by the addition of curcumin (Figure 5A). These results are in line with the observed decreased levels of CCL22 (Treg-attracting chemokine) upon treatment with curcumin and/or PIC (Figure 3).

### 2.6. The Effect of Curcumin on NF-κB Inhibition

We have shown before that macrophages are a main chemokine producer in the TME and are strong NF-κB activators [32]. In order to verify NF-κB inhibition in macrophages by curcumin, NF-κB nuclear translocation was examined via ELISA. Figure 5B shows a continuous increase of nuclear NF-κB concentration in macrophages treated with the positive control (PC) provided by the manufacturer or PIC after 1, 4, and 24 h. Macrophages treated with curcumin show no increase of nuclear NF-κB. Remarkably, coincubation with PIC and curcumin shows a reduction of nuclear NF-κB concentration compared to PIC treatment alone. This effect is visible at the time points of 4 and 24 h. After 1 h of coincubation, curcumin shows no effect on NF-κB activation by PIC.

These results were confirmed by measurement of NF-κB inhibitor alpha (IκBα) in the treated macrophages by Western blots. IκBα binds NF-κB in its inactivated form and prevents nuclear translocation. Phosphorylated IκBα (pIκBα) releases NF-κB, which is then able to translocate into the nucleus [24]. Compared to untreated macrophages, incubations with curcumin resulted in a similar appearance of IκBα but slightly lower pIκBα detection (Figure 5C), which indicates that NF-κB stayed in its inactivated form, located in the cytoplasm. PIC led to NF-κB activation, visible in almost no IκBα and high levels of pIκBα. Sendai virus, a dsRNA virus, was used as a positive control for activation of the TLR3 pathway. Accordingly, no visible IκBa levels and strong expression of pIκBα were observed. The combination of curcumin and PIC did not show a markable increase in IκBα but a decreased pIκBα detection compared to PIC alone, indicating the degradation of pIκBα and the prevention of nuclear translocation of NF-κB by curcumin.

### 2.7. NF-κB Inhibition by Curcumin Is More Potent Than Inhibition by BAY or CAPE

**Figure 6:**
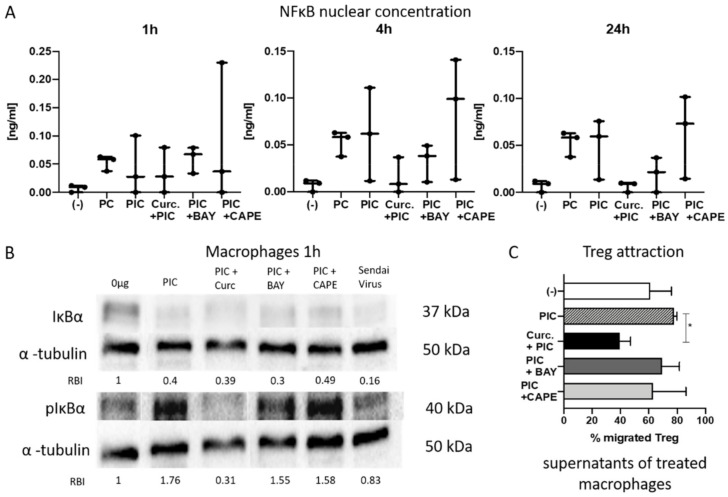
(**A**) Nuclear concentration of NF-κB in macrophages after incubation with adjuvants for 1, 4, and 24 h. Concentrations were determined by ELISA. Note that treatment with curcumin (Curc.) and PIC shows after 4 h less activation than the treatments with PIC and BAY or PIC and CAPE. (-) indicates the negative control. *n* = 3. Bars represent minimum to maximum with a line at the mean. (**B**) Western blot for IκBα, pIκBα, and α-tubulin after 1 h of macrophage treatment as indirect evidence for activated or inactivated NFκB. Relative band intensities (RBIs) were calculated between IĸBα and α-tubulin or pIĸBα and α-tubulin. The blot is representative for *n* = 3. Original blots can be found in Figure S1. (**C**) Treg attraction. Cancer specimens and macrophages were incubated with adjuvants for 24 h. After incubation, supernatants were harvested, and a migration assay was performed for 1.5 h. Flow cytometry was used to count migrated cells. *n* = 3 (PIC + BAY, PIC + CAPE), *n* = 4 ((-), PIC, Curc. + PIC). Bars represent mean with SEM. *P*-values were determined with the Mann–Whitney test, with * *p* < 0.05. The α-tubulin loading controls for IĸBα and pIĸBα shown in (**B**) are identical to the α-tubulin loading controls shown in Figure 5C since they are part of the same original blots; the figures are organized as shown to better align with the data persented in the results section.

BAY 11-7082 and CAPE are commonly known potent NF-κB inhibitors [36,37]. Therefore, we wanted to investigate if curcumin shows a significant advantage over these established inhibitors. The suppression of nuclear concentration of NF-κB and, therefore, nuclear translocation was measured by ELISA and found to be lower when macrophages were treated with PIC and BAY compared to PIC and curcumin. Similar to curcumin, the effects were visible after 4 and 24 h but not after 1 h. The combination of PIC and CAPE showed similar high nuclear NF-κB levels as with PIC alone (Figure 6A).

IκBa was present in untreated macrophages. As expected, a strong decrease was visible after treatment with PIC, but also after the combination of PIC with BAY, CAPE, or curcumin. The Sendai virus showed almost no IκBα signal. Accordingly, the detection of pIκBα was clearly visible for incubations with PIC, PIC and CAPE, PIC and BAY, and the Sendai virus. However, pIκBα detection for macrophages incubated with curcumin and PIC was barely noticeable (Figure 6B), indicating a faster degradation of pIκBα.

With regard to Treg migration towards tumor supernatants, incubation of curcumin with PIC resulted in a minimal number of migrated Tregs, while PIC and BAY, as well as PIC and CAPE, showed no differences compared to PIC treatment alone (Figure 6C).

## 3. Discussion

As several studies have shown in the past, EMT as a mechanism of tumor escape in the TME is a crucial factor in terms of tumor proliferation and metastasis [22,38]. Therefore, the reversal of EMT, in the form of a mesenchymal to epithelial transition, constitutes a promising therapeutic approach. In line with previous findings [21], we demonstrated that curcumin is able to transform EMT back to MET, with significantly decreased mesenchymal markers in cancer cell lines on the protein level as well as on the mRNA level. Furthermore, decreased gene expression levels of transcription factors like Twist and Snail, which are key factors in terms of EMT [25,39], were detected.

The balance of chemokines in the TME has an enormous impact on tumor progression and the inflammatory process as they decide upon a pro- or antitumor occurrence depending on which dominance of chemokines takes place. PIC is used as an adjuvant in combination with established therapies [35]. However, insufficient effects were observed due to NF-κB activation and the following CCL22 expression [32,40,41]. We have shown before that TLR3 induction results in an increased expression of CTLs, attracting chemokine CXCL10. However, a simultaneous increase of NF-κB-dependent CCL22 is visible, which results from TLR3 activation but also the activation of cytoplasmic helicases [32]. Using a selective TLR3 ligand, such as Rintatolimod, the negative effects can be decreased compared to treatment with PIC, a nonselective TLR3 ligand [42,43]. Rintatolimod is currently being tested in clinical studies (e.g., “Pembrolizumab, Cisplatin and Rintatolimod in treating Patients with recurrent ovarian, fallopian tube or primary peritoneal cancer”). However, each addition of an adjuvant to an already existing anticancer treatment can increase treatment-related side effects. A combination with curcumin, a natural product that selectively inhibits NF-κB, may be a welcomed alternative [31]. Referring to our analyzed cancer specimens, we showed that CCL22 expression was significantly decreased after coincubation of PIC with curcumin. Moreover, chemokine expression of CCL5 was increased in cancer specimens and macrophages; especially in macrophages, chemokine expression of CCL5 was significantly lower compared to PIC alone. There was no significant impact on CXCL10 in macrophage cultures, but there was a strong increase in the protein level of cancer specimens after coincubation with PIC and curcumin. The cause for this differential expression can be found in the different upstream signaling pathways of each chemokine. While CCL22 arises out of the TLR3-dependent NF-ĸB pathway and the cytoplasmic helicases [44] and CCL5 arise simultaneously out of the TLR3-dependent type-I interferon and NF-ĸB pathways [45], CXCL10 is exclusively generated via interferon I cascade [46]. This finding is attributable to the combined effect mechanism of PIC and curcumin. As our group has shown in the past, PIC as a synthetical dsRNA analog activates not only TLR3-dependent NF-κB and type-I interferon pathways but also TLR3-independent cytoplasmic helicases, which induce additional NF-κB activation [32]. In combination with the potential of curcumin to inhibit NF-κB, as we proved above, only the therapeutically welcomed type I signaling pathway remains activated. The nonsignificant decrease of CXCL10 in mRNA levels might be due to already-visible mRNA degradation and a lower rate of mRNA transcription compared to protein translation. In macrophage cultures, PIC led to a significant CCL5 increase, which is NF-κB-dependent and interferon-I-dependent [45], while in cancer specimens, a moderate expression was detected. In combination with curcumin, which inhibits NF-κB, a much lower CCL5 expression appears in macrophage cultures. These can be attributable to the aforementioned CCL5 NF-ĸB- and type-I interferon-dependent upstream signaling pathways. In contrast, no similar effects are visible with CXCL10, the production of which is only type-I interferon-dependent. Curcumin does not interfere with the interferon I pathway; therefore, no significant changes can be detected compared to the effects of PIC alone.

An important consequence of the predominance of certain chemokines is their capacity to attract Tregs or CTLs. They, in turn, determine a pro- or antitumor immune response. While an increased occurrence of Treg is noticed in the TME of most malignant cancers [47], a predominance of elevated CTL levels is associated with better tumor-specific immune response and superior patient outcomes [48]. CCL22 is a Treg-attracting chemokine [7], whereas CCL5 and CXCL10 are known to attract CTL [14]. For both cancer specimens and macrophages, we demonstrated significantly decreased Treg attraction upon incubation with curcumin alone, as well as curcumin in combination with PIC, which is in line with decreased CCL22 chemokine expression after combinatorial treatment. On the other hand, the increased expression of CTL-attracting chemokines CCL5 and CXCL10 may favor an enhanced antitumor immune response.

Previous studies demonstrated that curcumin inhibits NF-κB at different steps of the signaling cascade [29]. Inactivated NF-κB is linked to IκBα in the cytoplasm. By inducing phosphorylation of IκBα, NF-κB can dissociate and translocate into the nucleus to induce the transcription of multiple genes. At the same time, phosphorylated IκBα is degraded in the cytoplasm [24]. Intratumoral activation of NF-κB results in tumor progression and increased malignancy [25]. As a consequence, an inactivation of NF-κB and missing NF-κB-dependent production of tumor-promoting factors such as COX2, IDO, or interleukin 10 induction can provide new therapeutic opportunities and minimize negative side effects [28]. We were able to verify NF-κB inhibition indirectly by lower levels of phosphorylated IκBα after the combination of curcumin with PIC, whereas PIC alone led to increased levels of phosphorylated IκBα. Consequently, PIC leads to an activation of NF-κB via phosphorylation of IκB, whereas in combination with curcumin, NF-κB inhibition takes place. Results were confirmed by reduced detection of nuclear NF-κB concentration after coincubation of PIC with curcumin compared to PIC alone. NF-κB inhibition by curcumin takes place via inhibition of IκB kinases, which themselves lead to the phosphorylation of IκB and, thus, to NF-κB activation [29].

When comparing the NF-κB-inhibiting effects of curcumin with other known NF-κB inhibitors, we were able to show that curcumin induced the most potent NF-κB inhibition, resulting in decreased Treg migration. The inhibition of the NF-κB signaling pathway presents a potential therapeutic target. Both BAY and CAPE are known NF-κB inhibitors [36,37], with high levels of cytotoxicity [49,50,51]. Previous clinical trials have shown that not only the recommended daily doses of 3 mg/kg curcumin [52] were generally verified as safe, but, moreover, even doses of 12 g per day resulted in no significant side effects [53,54]. We compared the potential of NF-κB inhibition by curcumin, BAY, or CAPE to evaluate if curcumin’s effects are superior to these of BAY and CAPE and can, therefore, be considered a combinatorial adjuvant to already established therapies. We were thereby able to determine that the combination of CAPE with PIC did not affect the increased activation of NF-κB after 4 and 24 h of stimulation. BAY and PIC showed a slight effect towards reducing NF-κB expression, while the combination of PIC with curcumin led to decreased activation of NF-κB after 4 and 24 h. Both BAY and CAPE showed notable expression of pIκBα in macrophages, concomitant with NF-κB activation. While Treg attraction was reduced in the presence of PIC and curcumin, it was not influenced by the combination with BAY or CAPE. Taken together, we show that curcumin is a superior NF-κB inhibitor to BAY or CAPE.

Even though high doses of oral curcumin are well tolerated in patients, there is poor absorption in the gastrointestinal tract [55,56]. Transmucosal administration of a microgranular formulation is currently in evaluation [57]. Previous studies have shown that curcumin was able to enhance antitumor activity and reduce the negative side effects of tumor therapies (like dermatitis), while, at the same time, it is generally recognized as pharmacologically safe [55,58] We show that curcumin has great potential, particularly in the modulation of the TME. This study presents, for the first time, the immune-modulating effects of curcumin in HNSCCs, with potent inhibition of the Treg-attracting effects of PIC. We see further opportunities in curcumin as a therapeutic adjuvant for patients with increased NF-κB activation [59], e.g., patients with ovarian cancer [60], as well as in the prevention of recurrence of HNSCCs; curcumin also has potential as a prophylactic treatment in patients with recurrent dysplasia and positive smoking and alcohol history. Therefore curcumin is a promising drug in cancer therapy to supplement already established treatments. However, one limitation of this pilot study is the limited number of tumor samples, which will be increased in our future studies.

## 4. Materials and Methods

### 4.1. Cell Lines

Experiments have been performed with HNSCC cell lines UD (University of Düsseldorf; source Henning Bier, Düsseldorf, Germany)-SCC1 (RRID:CVCL_E324) and -SCC4 (RRID:CVCL_E327); the identity of the cell lines was proven by STR analysis. Cells were cultured in Dulbecco’s modified Eagle medium (DMEM, Gibco, 14190-094) supplemented with 10% fetal bovine serum (FBS) and 1% cell shield. Cultures were regularly examined for any possible contamination with mycoplasmas and found to be negative.

#### 4.1.1. Induction of Epithelial to Mesenchymal Transition

UDSCC1 and UDSCC4 cell lines were treated with StemXVivo EMT-inducing media supplement (R&D Systems, CCM017) according to the manufacturer’s protocol. For each cell line, 0.75 × 10^6^ cells were cultured in 75 cm^2^ flasks with 10 mL culture medium containing 100 µL EMT supplement (1×). On Day 3, after plating, media were replaced by fresh media containing StemXVivo EMT-inducing media supplement. On Day 5, cells were harvest for the following analyses.

#### 4.1.2. Incubation with Curcumin

Curcumin (Sigma Aldrich, Saint Louis, MO, USA, C7727) was dissolved in DMSO to a stock concentration of 100 µg/mL and used at indicated concentrations selected based on published data [61,62]. Native or EMT-induced cells were seeded in 24-well plates, with 1 × 10^6^ cells in 1 mL culture medium per well, and allowed to settle for 24 h. Then, cells were incubated for 48 h at 37 °C and 5% CO2 with 5, 10, 20, or 0 µg of curcumin per mL. To ensure equal DMSO amount in all treatment conditions, the final volume added to the cells was filled up to 20 µL with DMSO (i.e., for 20 µg/mL: 20 µL curcumin stock; for 10 µg/mL: 10 µL curcumin stock + 10 µL DMSO; for 5 µg/mL: 5 µL curcumin stock + 15 µL DMSO). Negative controls (0 µg/mL curcumin) were treated with 20 µL DMSO.

### 4.2. Patients

After informed, written consent, tumor specimens of treatment-naïve HNSCC patients were collected during primary tumor surgery at the Department of ENT, Head and Neck Surgery, at the University of Ulm. The study was approved by the local ethics committee (#19/15) and performed in accordance with the guidelines of the Declaration of Helsinki. The tumor biopsies were collected in the years 2019 and 2020. Additionally, the blood of healthy donors was processed for T-cell isolation as well as for monocyte isolation and macrophage differentiation.

#### Clinicopathological Characteristics of HNSCC Patients

The clinicopathological characteristics of HNSCC patients (*n* = 9) are listed in Table 1. The mean age was 56.5 years, with a range from 38 to 72 years. All patients did not receive any other cancer treatment before. Most patients were male (89%), and primary tumors were located in the oral cavity (22%), pharynx (45%), and larynx (33%). The majority of patients presented with advanced tumor stage T3/4 (78%); 89% had lymph node metastasis. Based on the Union for International Cancer Control (UICC) score, 45% were Stage I/II, and 55% were allocated to Stage II/IV; 55% of all patients consumed alcohol and/ or tobacco at the time of diagnosis.

### 4.3. Ex Vivo Tumor Tissue Explant Culture System

The ex vivo tumor tissue explant culture system was performed as previously described [32]. Briefly, tumor biopsies were transported to the laboratory, immediately after tumor resection, in physiological sodium chloride solution. Two 4-µm tumor punches per well were incubated in 24-well plates for 24 h in DMEM supplemented with 10 µg/mL curcumin +/− PIC (Sigma Aldrich, #p9582), 20 µg/mL PIC, 0.25 µg/mL CAPE (EMD Millipore, 211200) +/− PIC, 10 µmol/mL BAY 11-7082 (Sigma Aldrich, 196871) +/− PIC. The used concentrations of these adjuvants have been established before [63]**.**

The wells without treatment were incubated with 1 mL of DMEM. After 24 h, supernatants were collected for ELISA and migration assay, and tumor tissue was directly lysed to perform RNA-isolation for later RT-qPCR.

### 4.4. Annexin/PI Apoptosis Assay

To evaluate the cytotoxic effects of increasing Curcumin concentrations, native and EMT-induced cells were treated with different concentrations of curcumin, and apoptosis was assessed using the Annexin V-FITC Apoptosis Staining/Detection Kit (ab14085, Abcam) according to the manufacturer’s protocol. Briefly, cells were washed once with 1× annexin V binding buffer, provided with the kit, and resuspended in 500 µL annexin V binding buffer. Cells were stained with 5 µL Annexin V-FITC and 5 µl propidium iodide (PI) for 5 min at room temperature in the dark. Flow cytometry was performed with a Gallios flow cytometer (Beckman Coulter, Brea, CA, USA) and analyzed with Kaluza software 2.1.

### 4.5. Generation of Macrophages

For the generation of primary macrophages, Buffy coats from the blood bank (DRK Ulm, Ulm, Germany) were used. Monocytes were isolated from PBMCs by performing a positive selection using CD14 microBeads (Miltenyi Biotec, Auburn, CA, USA, 130-050-201). Cells were seeded at a concentration of 0.5 × 10^6^ cells/mL in 24-well plates (Thermo Fisher Scientific,Waltham, MA, USA, 142475) using IMDM medium. Macrophage differentiation was induced using 1000 U/mL granulocyte-macrophage colony-stimulating factor (GM-CSF) (Miltenyi Biotec, Auburn, CA, USA, 130-095-372). Half of the media was replaced on Day 3 with fresh IMDM media supplemented with 2× GM-CSF. On Day 6, the macrophages were ready for further use.

#### Incubation of Macrophages

Human, monocyte-derived macrophages were stimulated with either 10 µg/mL curcumin, 20 µg/mL PIC, 10 µg/mL curcumin and 20 µg/mL PIC, 20 µg/mL PIC and 10 µmol/L BAY 11-7082, 20 µg/mL PIC and 0.25 µg/mL CAPE, or 80 HAU/mL Sendai virus for 1, 4, and 24 h. Unstimulated controls were included. After the incubation period, the cells were used for mRNA isolation and Western blot and the supernatants for migration assays.

### 4.6. Western Blot

Cell lysates prepared from native and EMT-induced cells treated with different concentrations of curcumin, cell lysates prepared from untreated native and EMT-induced cells, as well as cell lysates prepared from macrophage cultures were incubated with different adjuvants, as mentioned above. Equal amounts of protein were mixed with 5× Laemmli buffer (BioRad #1610747, Hercules, CA, USA). Samples and 7 µL of protein ladder (BioRad) were loaded on precast protein gels (12%, BioRad, #4561044) and transferred to nitrocellulose membranes using the Trans-Blot Turbo RTA Transfer Kit (Bio Rad, #170-4270). Membranes were blocked with 5% milk in TBS-T or 5% BSA in TBS-T (vimentin detection) on a shaker for 1 h at RT. Membranes were incubated with primary antibodies E-cadherin (1:1000, BD, 610182), vimentin (1:1000, CST, 5741S), GAPDH (1:2000, Santa Cruz, sc-25778), IκBα (1:1000, CST, 9242S), pIκBα (1:1000, Invitrogen, #MA5-15224), or α-tubulin (1:1000, CST, #3873) overnight on a shaker at 4 °C. On the next day, membranes were washed with TBS-T and incubated with an HRP-conjugated secondary antibody (antirabbit, 1:10,000, 31460, Thermo Scientific, or antimouse, 1:10,000, 31450, Thermo Scientific) on a shaker for 1 h at RT. Detection of chemiluminescence was performed with the Super Signal West Dura Extended Duration Kit (Thermo Scientific, 34075), and, for densitometric analysis, Image Lab Software (Bio Rad) was used.

### 4.7. Flow Cytometry

E-cadherin and vimentin levels were evaluated by flow cytometry. Native and EMT-induced cells, treated with different concentrations of curcumin, were stained with FITC-conjugated anti-E-cadherin (Thermo Fisher Scientific, Waltham, MA, USA, 11-0219-42) or Alexa Flour-488-conjugated anti-vimentin (BD 560081) for 30 min at 4 °C in the dark. For the intracellular staining of vimentin, cells were prepared with Cytofix/Cytoperm (BD, 10482735) in advance, according to the manufacturer’s protocol. Flow cytometry was performed with a Gallios flow cytometer (Beckman Coulter) and analyzed with Kaluza software 2.1.

### 4.8. ELISA

ELISA was performed with supernatants of macrophage and tumor tissue cultures after 24 h of incubation, as previously described [32]. EIA plates (Corning, #3590) were coated with 1 µg/mL of specific capture antibody for each chemokine (R&D Systems, Minneapolis, MN, USA, CCL5: MAB678; CXCL10: MAB266; CCL22: MAB336) and incubated for 24 h at 4 °C. Standards were diluted in blocking buffer in a range of 20–0.156 ng/mL (R&D Systems, CCL5: 278-RN-010; CXCL10: 266-IP-010; CCL22: 336-MD-025). After 2 h of blocking, plates were washed three times with wash buffer; 50 µL of standards and nondiluted samples were added and incubated for 1.5 h at RT. Plates were washed three times and incubated with 0.25 µg/mL specific biotinylated detection antibodies (R&D Systems, CCL5: BAF278; CXCL10: BAF266; CCL22: BAF336) for 1 h at RT. After washing, 100 µL HRP-conjugated streptavidin (Thermo Scientific, Cat. #N100), diluted 1:10,000 in ELISA blocking buffer, was added and incubated for 20 min at RT. Plates were washed, and 100 µL of TMB (Thermo Scientific, Cat. #N301) was added to induce color development. After 6 min, samples were read in TECAN infinite M2000 Pro using Magellan software at 450 nm, with 0.1 s per well timing.

### 4.9. NF-κB ELISA

NF-κB ELISA for evaluation of NF-κB nuclear translocation was performed according to the manufacturer’s protocol using the TransAM^®^ NF-κB p65 Activation Assay (Active Motif #40096, Carlsbad, CA, USA).

Briefly, pretreated cells were washed with ice-cold PBS/PIB, and cell scrapers were used to detach cells from the surface. After cell lysis, supernatants containing the cytoplasmatic fraction were removed, and the nuclear pellet was resuspended in complete lysis buffer. The supernatants containing the nuclear fraction were saved and placed in a 96-well plate with complete binding buffer. After 1 h incubation, the NF-κB antibody (1:1000 dilution in 1× antibody binding buffer) was added to each well, followed by incubation for 1 h. Following treatment with the HRP-conjugated antibody, a developing solution was added, and the reaction stopped after 5 min. Samples were measured at 450 nm using TECAN infinite M2000 Pro and Magellan software.

### 4.10. mRNA Isolation

RNA isolation was performed according to the manufacturer’s protocol using the RNeasy^®^ Mini Kit (50; Qiagen 74104). Tumor punches were lysed in 2 mL microtubes containing a steal ball and 600 µL RLT supplemented with ß-mercaptoethanol (1:100) on a tissue lyser. Macrophages were directly harvested by adding 700 µL RLT to each well. Subsequently, samples were added to an RNeasy Mini spin column and placed into a 2-mL collection tube and centrifuged for 15 s at 8000G; the flow-through was discarded. Afterward, 350 µL RW1 buffer was added to each column, and samples were centrifuged for another 15 s at 8000G. Then, 80 µL of DNase incubation mix was added to each column before incubating for 15 min at RT. Next, 350 µL of RW1 buffer was added to each sample, and another centrifugation step proceeded for 15 s at 8000G; 500 µL RPE buffer was added twice, and samples were centrifuged 15 s at 8000G. Columns were placed in fresh microtubes, and 50 µL of RNase-free water was added to each sample before centrifuging for 1 min at 8000G. RNA concentration was determined using a TECAN spectrophotometer.

### 4.11. cDNA Synthesis and RT qPCR

cDNA was synthesized by using the Quanti Nova RT Kit (Qiagen, 205413) according to the manufacturer’s protocol. Briefly, 2 µL gDNA removal mix was mixed with RNase-free water and 500 ng template RNA in PCR tubes, resulting in a final volume of 15 µL. The reaction mix was placed in a thermocycler for incubation for 2 min at 45 °C. Tubes were placed on ice, and 4 µL of reverse transcription mix, together with 1 µL of reverse transcription enzyme, was added. Reverse transcription was then performed in a thermocycler, with 3 min at 25 °C, 10 min at 45 °C, and 5 min at 85 °C.

RT qPCR was performed using the Quanti Nova SYBR Green PCR Kit (Qiagen, 208056). First, a reaction mix was prepared by mixing 10 µL of 2x SYBR Green PCR Master Mix, 0.6 µL Primer A and 0.6 µL Primer B or 1 µL PrimerMix and RNase-free water, resulting in a final volume of 18 µL. Then, 2 µL cDNA was added, and RT qPCR was run in a Roche Light Cycler using the following program: 2 min of initial heat activation at 95 °C, followed by 2-step cycling of 5 s denaturation at 95 °C and 10 s combined annealing and extension at 60 °C, repeated for 35–40 times. Primers are listed in Appendix A Table A1.

Experiments were performed in duplicate, and hypoxanthine phosphoribosyl transferase (HPRT) was used as the normalization control. The Delta Ct value (∆Ct) was calculated between the target and the HPRT mean of the same condition. Relative target mRNA expression levels compared to HPRT were calculated using the 2−∆Ct method.

### 4.12. Migration Assay

Peripheral blood mononuclear cells (PBMCs) from Buffy coats were isolated by density gradient centrifugation with Leukosep columns (Greener) and Biocoll Separating Solution (Biochrom) and used for CD4+CD39+ Treg isolation, as we have performed before [64,65]. The CD4^+^ T-Cell Isolation Kit (Miltenyi Biotec, Auburn, CA, USA, 130-096-533), anti-Biotin MicroBeads (Miltenyi Biotec, 130-090-485), and anti-CD39 MicroBeads (Miltenyi Biotec, 130-100-459) were applied according to the manufacturer’s protocol. The purity of the isolated Treg populations was checked by flow cytometry using antibodies against surface markers CD39 and CD4 and was greater than 95%. Supernatants from macrophage or ex vivo tumor tissue cultures, after 24 h of co-incubation with adjuvants, were used as Treg attraction medium. Treg and supernatants were prewarmed to 37 °C for 15–20 min; 500 µL of supernatants were gently dropped into the bottom well of a 5-µm pore size Transwell plate (Costar, #3421, Kennebunk, ME, USA). Next, 200 µL of Treg suspension was added to the upper well. After 1.5 h of incubation, inserts were carefully removed and discarded. Migrated cells in the bottom well were harvested, and flow cytometry was used to count migrated cells by gating on viable cells and doing a 1.5 min run for each sample.

### 4.13. Statistics

Statistical analysis was performed using Prism version 8 (Graphpad, San Diego, CA, USA). Box and whiskers graphs depict the median as a horizontal line, boxes display the interquartile range, and whiskers extend from the minimal to the maximal data point. Column bar graphs represent means with standard deviation (SD). As a nonparametric test of unpaired values, the Mann–Whitney test was chosen. A *p*-value of <0.05 was used to evaluate the significance of the data.

## 5. Conclusions

Curcumin is a potent NF-κB inhibitor that is able to reverse the process of EMT back to MET, reducing Treg-attracting chemokine CCL22, with visible inhibition of Treg migration. Additionally, only coincubation of PIC with curcumin and not with other NF-κB inhibitors resulted in an inhibition of PIC-dependent NF-κB activation, with an unhindered activation of the therapeutically desired type-I interferon signaling pathway. Hence, this study indicates that curcumin can intensify the beneficial qualities of PIC by suppressing its negative properties by NF-κB inhibition. Curcumin has the potential as a safe adjuvant for combinatorial treatments, not only with PIC but probably also with other potent NF-κB activators, such as other ligands of the TLR family.

## Figures and Tables

**Table 1:** Patient characteristics.

Characteristics		Patients (*n* = 9)
	*n*	%
**Age**		
≥65	7	77
≤65	2	23
**Gender**		
Male	8	89
Female	1	11
**Primary tumor site**		
Oral cavity	2	22
Pharynx	4	45
Larynx	3	33
**Tumor stage**		
T1	1	11
T2	1	11
T3	6	67
T4	1	11
**Nodal status**		
N0	1	11
N+	8	89
**Distant metastasis**		
M0	9	100
**UICC stage**		
I/II	4	45
III/IV	5	55
**HPV status**		
(p16 +/HPV-DNA+)		
Positive	1	11
Negative	3	33
Undefined	5	55
**Alcohol consumption**		
Yes	8	89
No	1	11
**Tobacco consumption**		
Yes	5	55
No	4	45

## Data Availability

The data presented in this study are available on request from the corresponding author.

## Supplementary Materials

The following are available online at https://www.mdpi.com/2072-6694/13/6/1335/s1, Figure S1: Original blots for Western blots used in Figure 1, Figure 5 and Figure 6 of the manuscript.

## Abbreviation

Annexin/PI	annexin/propidium iodide
CAPE	caffeic acid phenethyl ester
CTL	cytotoxic T-cell
COX2	cyclooxygenase 2
DC	dendritic cell
EMT	epithelial to mesenchymal transition
FBS	fetal bovine serum
GM-CSF	granulocyte-macrophage colony-stimulating factor
HNSCC	head and neck squamous cell carcinoma
IDOI	indolamin-2,3-dioxygenase
κBα	NFκB inhibitor, alpha
MDSC	myeloid-derived suppressor cell
MET	mesenchymal to epithelial transition
NFκB	nuclear factor kappa of activated B-cells
PBMCs	peripheral blood mononuclear cells
PIC	polyinosinic:polycytidylic acid
pIκBα	phosphorylated nuclear factor of kappa light polypeptide gene enhancer in B-cells inhibitor, alpha
RT	room temperature
Treg	regulatory T-cells
TME	tumor microenvironment
TRL3	toll-like receptor 3
UDSCC	University of Düsseldorf squamous cell carcinoma
UICC	Union for International Cancer Control

## Appendix A

**Table A1:** PCR Primers.

**Primer Names**	**Primer Sequences**	**Manufacturer**
Vimentin—For	5′- GAGAACTTTGCCGTTGAAGC-3′	Biomers
Vimentin—Rev	5′- GCTTCCTGTAGGTGGCAATC-3′	Biomers
SNAIL—For	5′- TCGGAAGCCTAACTACAGCGA-3′	Biomers
SNAIL—Rev	5′- AGATGAGCATTGGCAGCGAG-3′	Biomers
TWIST—For	5′- GGAGTCCGCAGTCTTACGAG-3′	Biomers
TWIST—Rev	5′- TCTGGAGGACCTGGTAGAGG-3′	Biomers
**Primer Names**	**Unique Assay ID**	**Manufacturer**
CCL5	qHsaCID0011644	Bio Rad
CXCL10	qHsaCED0046619	Bio Rad
CCL22	qHsaCID0015408	Bio Rad
