# Supplementary material for: Immune-Stimulatory Effects of Curcumin on the Tumor Microenvironment in Head and Neck Squamous Cell Carcinoma
Source: Cancers (Basel). 2021 Mar 16;13(6):1335. doi: 10.3390/cancers13061335 (PMC8001767; doi:10.3390/cancers13061335)
Supplement: Supplementary file 1 [file cancers-13-01335-s001.zip › cancers-1100015-supplementary.pptx]

## Slide 1
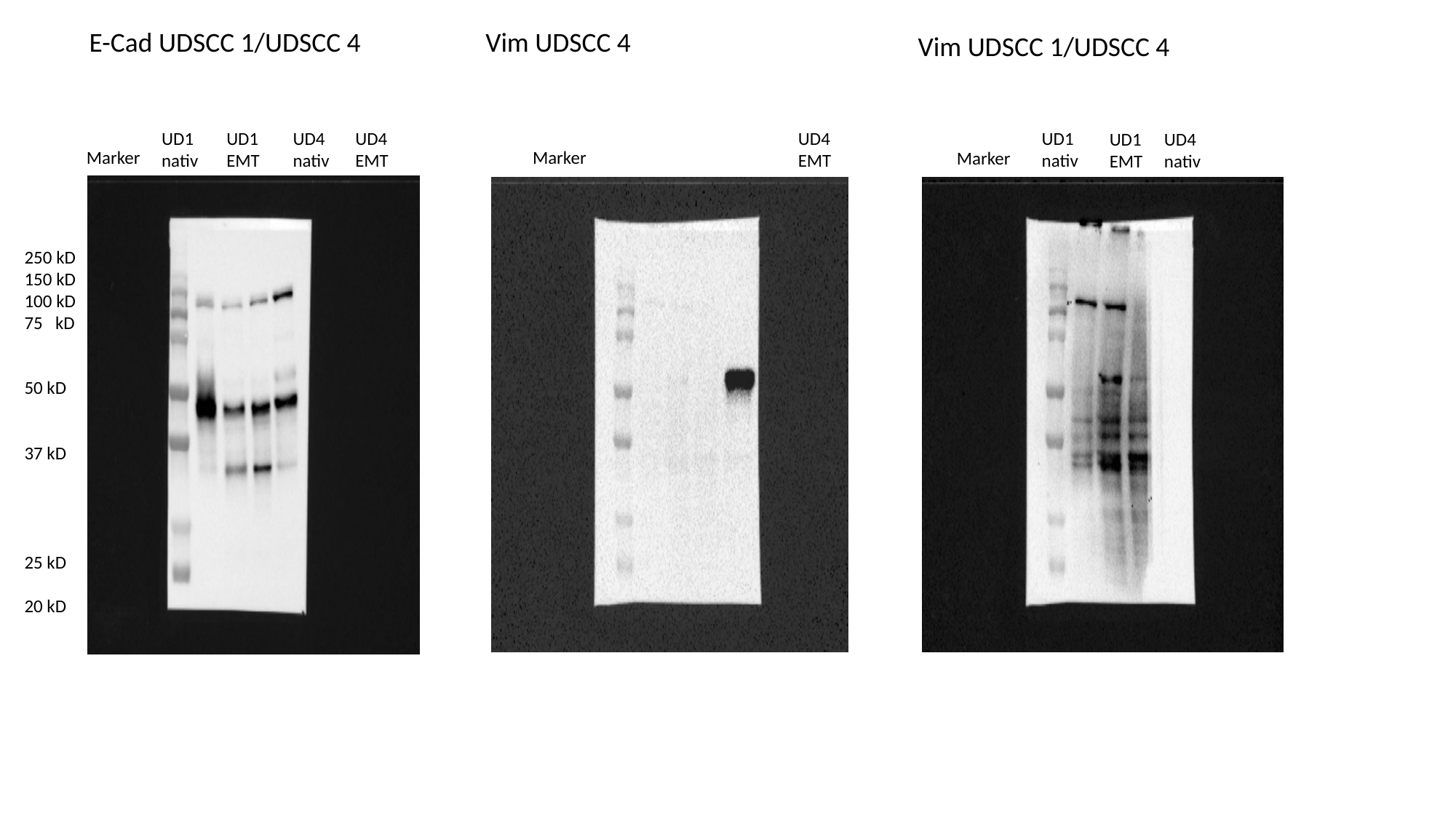

Vim UDSCC 4
E-Cad UDSCC 1/UDSCC 4
Vim UDSCC 1/UDSCC 4
UD4
nativ
UD4
EMT
UD1
EMT
UD1
nativ
Marker
UD4
EMT
UD1
nativ
UD1
EMT
UD4
nativ
Marker
Marker
250 kD
150 kD
100 kD
75 kD
50 kD
37 kD
25 kD
20 kD
#

## Slide 2
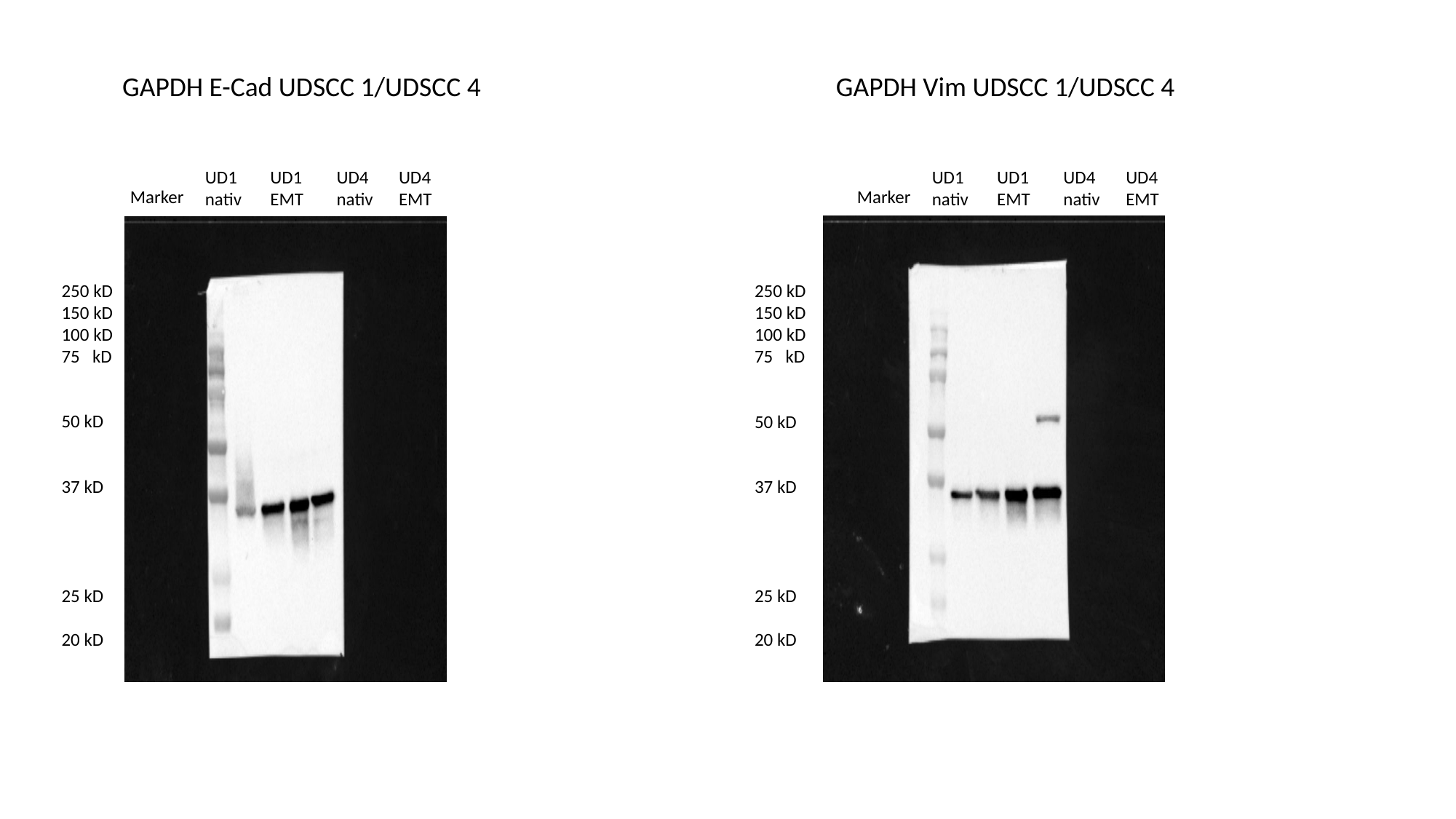

GAPDH E-Cad UDSCC 1/UDSCC 4
GAPDH Vim UDSCC 1/UDSCC 4
UD4
nativ
UD4
EMT
UD1
EMT
UD1
nativ
Marker
UD4
nativ
UD4
EMT
UD1
EMT
UD1
nativ
Marker
250 kD
150 kD
100 kD
75 kD
50 kD
37 kD
25 kD
20 kD
250 kD
150 kD
100 kD
75 kD
50 kD
37 kD
25 kD
20 kD

## Slide 3
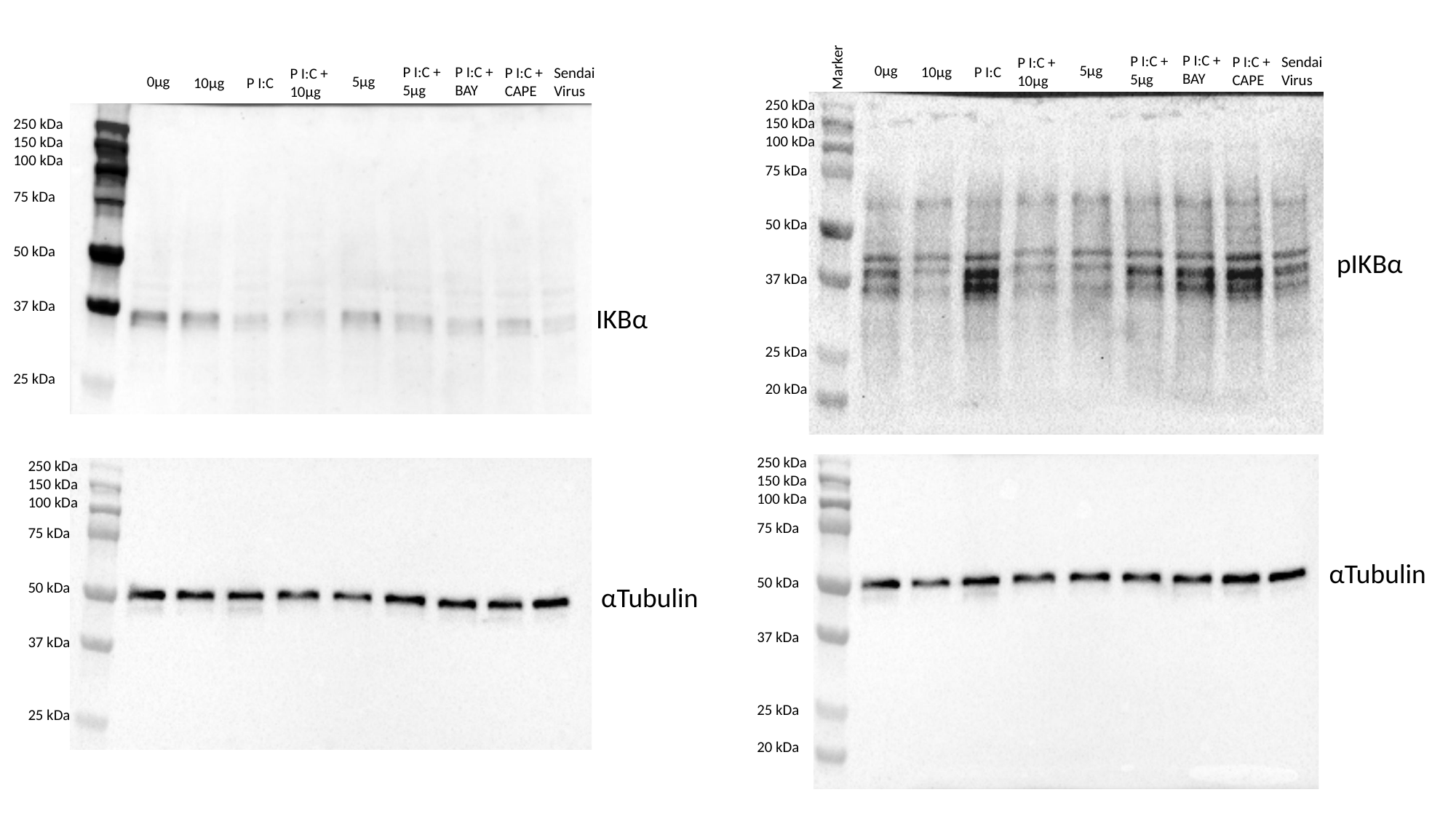

Marker
P I:C +
BAY
P I:C +
5µg
P I:C +
CAPE
Sendai
Virus
P I:C +
10µg
0µg
5µg
P I:C
10µg
250 kDa
150 kDa
100 kDa
75 kDa
50 kDa
37 kDa
25 kDa
20 kDa
pIKBα
250 kDa
150 kDa
100 kDa
75 kDa
50 kDa
37 kDa
25 kDa
20 kDa
αTubulin
P I:C +
BAY
P I:C +
5µg
P I:C +
CAPE
Sendai
Virus
P I:C +
10µg
0µg
5µg
P I:C
10µg
250 kDa
150 kDa
100 kDa
75 kDa
50 kDa
37 kDa
25 kDa
IKBα
250 kDa
150 kDa
100 kDa
75 kDa
50 kDa
37 kDa
25 kDa
αTubulin
